# Investigating the molecular basis of multiple insecticide resistance in a major malaria vector *Anopheles funestus* (*sensu stricto*) from Akaka-Remo, Ogun State, Nigeria

**DOI:** 10.1186/s13071-020-04296-8

**Published:** 2020-08-18

**Authors:** Seun M. Atoyebi, Genevieve M. Tchigossou, Romaric Akoton, Jacob M. Riveron, Helen Irving, Gareth Weedall, Eric Tossou, Innocent Djegbe, Isaac O. Oyewole, Adekunle A. Bakare, Charles S. Wondji, Rousseau Djouaka

**Affiliations:** 1 International Institute of Tropical Agriculture, 08 BP 0932, Cotonou, Benin; 2 Cell Biology & Genetics Unit, Department of Zoology, University of Ibadan, Ibadan, Nigeria; 3 University of Abomey Calavi, BP 526, Cotonou, Benin; 4 Liverpool School of Tropical Medicine, Pembroke Place, Liverpool, L3 5QA UK; 5 Insecticide Bioscience Department, Syngenta, Toulouse, UK; 6 Liverpool John Moores University, Liverpool, L3 3AF UK; 7 National University of Sciences, Technologies, Engineering and Mathematics, Ecole Normale Supérieure de Natitingou, BP 123, Natitingou, Benin; 8 Biology Department, Babcock University, Ilisan Remo, Ogun State Nigeria; 9 Centre for Research in Infectious Diseases (CRID), Yaounde, Cameroon

**Keywords:** *Anopheles funestus*, Permethrin, DDT, Metabolic genes, Insecticide Resistance mechanisms, Nigeria

## Abstract

**Background:**

Understanding the mechanisms used by *Anopheles* mosquitoes to survive insecticide exposure is key to manage existing insecticide resistance and develop more suitable insecticide-based malaria vector control interventions as well as other alternative integrated tools. To this regard, the molecular basis of permethrin, DDT and dieldrin resistance in *Anopheles funestus* (*sensu stricto*) at Akaka-Remo was investigated.

**Methods:**

Bioassays were conducted on 3–5-day-old adult *An. funestus* (*s.s.*) mosquitoes for permethrin, DDT and dieldrin susceptibility test. The molecular mechanisms of mosquito resistance to these insecticides were investigated using microarray and reverse transcriptase PCR techniques. The voltage-gated sodium channel region of mosquitoes was also screened for the presence of knockdown resistance mutations (*kdr* west and east) by sequencing method.

**Results:**

*Anopheles funestus* (*s.s.*) population was resistant to permethrin (mortality rate of 68%), DDT (mortality rate of 10%) and dieldrin (mortality rate of 8%) insecticides. Microarray and RT-PCR analyses revealed the overexpression of glutathione S-transferase genes, cytochrome P450s, esterase, trypsin and cuticle proteins in resistant mosquitoes compared to control. The *GSTe2* was the most upregulated detoxification gene in permethrin-resistant (FC = 44.89), DDT-resistant (FC = 57.39) and dieldrin-resistant (FC = 41.10) mosquitoes compared to control population (FC = 22.34). The cytochrome P450 gene, *CYP6P9b* was also upregulated in both permethrin- and DDT-resistant mosquitoes. The digestive enzyme, trypsin (hydrolytic processes) and the cuticle proteins (inducing cuticle thickening leading to reduced insecticides penetration) also showed high involvement in insecticide resistance, through their overexpression in resistant mosquitoes compared to control. The *kdr* east and west were absent in all mosquitoes analysed, suggesting their non-involvement in the observed mosquito resistance.

**Conclusions:**

The upregulation of metabolic genes, especially the *GSTe2* and trypsin, as well as the cuticle proteins is driving insecticide resistance of *An. funestus* (*s.s.*) population. However, additional molecular analyses, including functional metabolic assays of these genes as well as screening for a possible higher cuticular hydrocarbon and lipid contents, and increased procuticle thickness in resistant mosquitoes are needed to further describe their distinct roles in mosquito resistance.
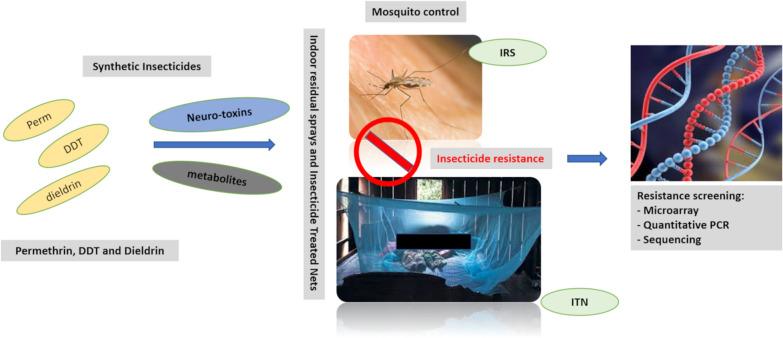

## Background

A population of *Anopheles funestus* (*sensu stricto*) in Nigeria was recently shown to be resistant to different classes of public health insecticides commonly used for malaria vector control [1]. There was a higher concern with permethrin resistance (68% mortality) at Akaka-Remo due to the over-reliance on pyrethroids for insecticide-treated nets (ITNs) and a few indoor residual sprays (IRS) [2]. DDT (10% mortality) and dieldrin (8%) resistance reported is also delaying the re-introduction of these cheap insecticides as alternatives to pyrethroids [1]. There is an increasing rate of insecticide resistance of *An. funestus* (*sensu lato*) in Africa and resistance has been observed to have spread across different regions [3–5]. One of the main concerns from existing reports is that each population displays its peculiar mechanisms to tolerate insecticides exposure [6–8]. Nevertheless, populations within the same region may share similar resistance mechanisms, which might be a consequence of gene flow across these regions [6].

To date, metabolic-based mechanisms have been the main driver of insecticide resistance in *An. funestus* (*s.l.*) [5–8]. Cytochrome P450s (P450s) and glutathione S-transferase (GSTs) are two gene families that are always associated with the mosquito resistance. The P450s such as *CYP6P9a/b*, *CYP6M7*, *CYP9K1* and *CYP6Z1* have various roles they play in pyrethroids resistance [4, 9]. Interestingly, as resistance gets stronger, more P450 genes are evolving in resistant mosquitoes [4, 8]. Glutathione S-transferases, a phase II detoxification gene family are known to be involved in both permethrin and DDT resistance of *An. funestus* (*s.l.*). Several reports have shown that the epsilon class, *GSTe2* is a key detoxification gene, which is displayed by its overexpression in permethrin and DDT-resistant mosquitoes [10, 11]. In addition to its overexpression, the nucleotide substitution process on position 119 on the gene, thereby changing leucine to phenylalanine has given the *GSTe2* a greater advantage to metabolise DDT [11].

Djouaka et al. [1] have already reported the roles of oxidase and high resistant allelic frequency (f(R) = 77%) of the L119F-GSTe2 mutation in resistant *An. funestus* at Akaka-Remo. However, there is a need for thorough molecular-based analyses to further understand the various mechanisms developed by the *An. funestus* (*s.s.*) population against insecticides. For these analyses, a genome-wide transcriptional analysis by microarray and the reverse transcriptase polymerase chain reaction (RT-PCR) were conducted to identify the set of detoxification genes associated with DDT and permethrin resistance in the mosquito population. Also, the voltage-gated sodium channel (VGSC) sequence (permethrin and DDT target site) was screened to investigate the presence of knockdown resistance (*kdr*) mutations (L1014F and L1014S) in wild (F_0_) mosquito species.

## Methods

This is a follow-up study from Djouaka et al. [1]. From the previous study, *An. funestus* (*s.s.*) collected were exposed to permethrin, deltamethrin, DDT, dieldrin, bendiocarb and malathion, with bioassay test showing that this mosquito population was resistant to all insecticides tested except for malathion. The F_1_ mosquitoes generated from this previous study were further analysed for their molecular mechanisms of resistance in the present study. Below is a brief methodology on how mosquitoes were collected, and bioassays were conducted, already documented in the previous paper (for details, see Djouaka et al. [1]).

### Description of field mosquitoes and the laboratory susceptible strain, FANG

Female blood-fed adult *An. funestus* (*s.l.*) mosquitoes were collected indoors between 6:00 and 10:00 h at Akaka-Remo (6°57′N, 3°43′E) using electric aspirators. Field mosquito samples were collected from October 2014 to April 2015. After collections, mosquitoes were sorted out by species and the blood-fed were kept into cups until fully gravid. Thereafter, they were forced to lay eggs [12] that were sent *via* courier to the Liverpool School of Tropical Medicine (LSTM) for rearing into F_1_, insecticide susceptibility tests [13] and molecular analysis. *Anopheles funestus* (*s.l.*) were first identified morphologically using standard keys [14] and later identified molecularly [15] to the subspecies level. Hatched eggs were pooled and reared together (temperature of 25–28 °C and relative humidity of 80%) in mineral water, which was renewed every two days to reduce mortality, and the resulting larvae were daily fed with Tetramin™ baby fish.

For laboratory experiments, mosquito populations used were defined as: (i) permethrin-resistant, also referred to as Rperm: *An. funestus* that were resistant after exposure to 0.75% permethrin; (ii) DDT-resistant, also referred to as R_DDT_: *An. funestus* that were resistant after exposure to 4% DDT; (iii) unexposed mosquitoes, also referred to as Control and represented with the symbol C: *An. funestus* that were not exposed to any insecticide and used as control samples during bioassay and other analysis; (iv) fully susceptible *An. funestus* strain, also referred to as FANG or susceptible mosquitoes and represented with the symbol S: FANG mosquitoes are insecticide-susceptible strain of *An. funestus*, that originated from southern Angola and have been maintained in the laboratory since January 2003 [16, 17].

### Bioassays and synergist test with DEM and DEF

The 3–5-day old F_1_ adult samples obtained from pooled mosquitoes were exposed to permethrin (0.75%), DDT (4%) and dieldrin (4%) to assess the insecticide susceptibility status of *An. funestus* (*s.s.*) population. A minimum of 100 F_1_ mosquito samples were tested for each insecticide according to the WHO guidelines [13]. At least 4 replicates of 25 mosquitoes per tube were exposed to insecticide-impregnated papers (with 2 replicates of 25 mosquitoes exposed to untreated papers as control) for 1 h and were immediately transferred into clean holding tubes with 10% sugar solution. Mortality records were taken after 24 h post-exposure as described by the WHO [13]. Resistant and susceptible mosquitoes generated from bioassays were stored in pools of 10 and 5, respectively at − 80 °C for molecular analysis [18].

Furthermore, another set of mosquitoes were used for the synergist test to analyse the roles of GSTs and esterase in the observed resistance. Mosquitoes were first exposed to diethyl maleate (DEM) and S,S,S-tributyl phosphorotrithioate (DEF) for 1 h each and were immediately exposed to either permethrin or DDT for another 1 h according to the WHO guidelines [13] to evaluate the roles of glutathione S-transferase (DEM) and esterase (DEF) in the observed phenotypic resistance. This mosquito population has been previously exposed to PBO to evaluate the role of oxidase and results are available in Djouaka et al. [1]. At least 50 mosquitoes were tested, and mortality was recorded 24 h post-exposure [13].

### Microarray

The genome-wide transcriptional screening was done to identify gene sets that were differentially overexpressed in resistant *An. funestus* mosquitoes at Akaka-Remo, as a possible mechanism for the observed phenotypic resistance. The Agilent *Anopheles funestus* chip, 8 × 60k (60-mer) designed using the eArray program (Agilent: A-MEXP-2374) was used for the microarray hybridization analysis. This chip contains the 4 × 44 array (A-MEXP-2374) [10] plus an additional 15,527 expressed sequence tags (ESTs) generated from a transcriptome sequence analysis of *An. funestus* [19]. Overall, each array is incorporated with 60-mer probes designed from 8540 ESTs (2 probes for each EST) generated from *An. funestus* transcriptome 454 sequencing [20], a set of 2850 *An. funestus* cDNAs from GenBank (2 probes for each EST), a set of P450 genes (3 probes for each gene) from the *rp1* and *rp2* QTL BAC sequence [21, 22], and the 13,000 transcripts of the complete *An. gambiae* genome. Also, all of the *An. gambiae* detoxification genes present on the *An. gambiae* detox chip [23] were added to this chip with 3 probes for each gene to explore all possible gene sequences conserved between *An. gambiae* and *An. funestus*.

Total RNA was extracted from three batches of a pool of 10 *An. funestus* (*s.s.*) mosquitoes in the different populations using Picopure RNA Isolation Kit (Arcturus, Waltham, USA). The quantity and quality of RNA were assessed using NanoDrop ND1000 spectrophotometer (Thermo Fisher Scientific, Waltham, MA, USA) and Bioanalyzer (Agilent, Santa Clara, CA, USA), respectively. The complementary RNA (cRNA) was then amplified from each extracted RNA by labelling resistant samples (R) with cy5 dye and susceptible strain FANG samples (S) with cy3 dye using the Agilent Quick Amp Labeling Kit (two-color) following the manufacturer’s protocol. The quality and quantity of the labelled cRNA samples were further assessed as described above before hybridization on the array at 65 °C for 17 h. In total, five and four hybridizations were done for permethrin and DDT comparisons, respectively, which consist of three (permethrin) and two (DDT) biological replicates, as well as two dye swaps each.

### Reverse transcriptase PCR analysis

The expression pattern of known resistant-associated genes was evaluated in resistant samples compared to the unexposed control using the RT-PCR. This analysis was performed for some selected metabolic genes that were either identified in the microarray analysis or previously associated with permethrin and DDT resistance in *An. funestus* mosquitoes [8, 11, 20, 21]. Metabolic genes analysed are glutathione S-transferase family (*GSTe2*, *GSTd3*, *GSTd1-5*); cytochrome P450 genes (*CYP6P9a*, *CYP6P9b*, *CYP6P4a*, *CYP6P4b*, *CYP6M7*, *CYP6AA1*, *CYP4C27*, *CYP9K1*); aldehyde oxidase (*Ald oxi*) and trypsin. This experiment was conducted using three batches of a pool of 10 resistant R_perm_ and R_DDT_, unexposed control and FANG mosquitoes [10]. Briefly, RNA extracted from each pool was used to synthesize cDNA using Superscript III (Invitrogen, Waltham, USA) with oligodT20 and RNase H according to the manufacturer’s instructions. The RT-PCR amplification was performed using the MX 3005P (Agilent, California, USA) system: first, five-serial dilutions of the synthesize cDNA samples were done and analysed to generate PCR efficiency and quantitative differences between samples, where a standard curve was generated for each gene. The following PCR mix was used for a single reaction in this experiment: 10 μl SyBr Green; 0.6 μl of both the forward and reverse 10 mM primers (Table 1); 7.8 μl dH_2_O; 1 μl cDNA template. Also, thermal condition parameters used are: 1 cycle at 90 °C for 3 min for initial denaturation, 40 cycles of 95 °C for 10 s and 60 °C for 10 s for the amplification (final denaturation, annealing, extension and fluorescence read), and 1 cycle of 95 °C for 1 min, 55 °C for 30 s and 95 °C for 30 s for dissociation/melting. The primers used for target genes are attached (Table 1).

**Table 1:** List of primers used for the reverse transcriptase PCR

Gene primer	Forward (5′-3′)	Reverse (5′-3′)
CYP6M7	CCAGATACTGAAAGAGAGCCTTCG	CAAGCACTGTCTTCGTACCG
CYP6P9a	CAGCGCGTACACCAGATTGTGTAA	TCACAATTTTTCCACCTTCAAGTAATTACCCGC
CYP6P9b	CAGCGCGTACACCAGATTGTGTAA	TTACACCTTTTCTACCTTCAAGTAATTACCCGC
CYP6P4a	AACTCGTATTCGACCCCAAA	CGTTTCCATGGAATTACATTTTCTG
CYP6P4b	AACTCGTATTCGACCCCAAA	ACAATCATTATACCACACATCTGAC
CYP6AA4	CATCTGGCTGAATGGCACTA	TCAACAATGCCATCAAATCG
CYP9K1	AGGGCTTCTGGATACGGTTC	CGTACGGTTCGGTTTTGATT
Trypsin	GGCCACAACCTCAAAGTCTC	CGACAGAAATCAGTCGTTAGTACG
GSTe2	GTTTGAAGCAGTTGCCATACTACGAGG	TCAAGCTTTAGCATTTTCCTCCTTTTTGGC
GSTd3	CACGGCCAGTCCTCTTTTAG	AAGCTTCTTCGCCACCAGTA
GSTd1-5	TGGAGAAATACGGCAAGGAC	CTTGGCGAAGATTTGTGGAT
Aldehyde oxidase	GCTCTGAACATTGCACCTCA	TGGTGTCGAACGATTGTGTT
RSP7	GTGTTCGGTTCCAAGGTGAT	TCCGAGTTCATTTCCAGCTC
Actin	TTAAACCCAAAAGCCAATCG	ACCGGATGCATACAGTGACA

### Partial sequencing of the VGSC gene, target site of permethrin and DDT

A fragment spanning a portion of the voltage-gated sodium channel gene (VGSC), containing the 1014 codon associated with insecticide resistance in *An. gambiae* was amplified in ten wild female *An. funestus* mosquitoes from Akaka-Remo using the KdrFunR2 primer (5′-CCG AAA TTT GAC AAA AGC AAA-3′) [6, 18, 24]. The amplicons were purified using the Qiaquick purification kit (Qiagen, Hilden, Germany) before subjecting to sequencing.

### Data analysis

After hybridization and scanning in the microarray experiment, data obtained were analyzed using the Genespring GX 13.0 software: differentially expressed genes were selected at a statistical significance level of *P* ≤ 0.05 with Benjamini-Hochberg correction for multiple testing of a cut-off of 2 fold change (FC), except for some exemptions (comparisons of DDT-S and Perm-C) that differentially expressed genes were only identified at no correction multiple testing but still with t-test against zero and a cut-off of 1.5 FC at *P* ≤ 0.05.

For the reverse transcriptase PCR analysis, resulting data for each gene was normalized with housekeeping genes: ribosomal protein S7 (RSP7; AGAP010592), and actin 5C (AGAP000651) before calculating the relative expression level and FC of each target gene in resistant and control relative to the susceptible according to the 2-ΔΔCQ method, incorporating the PCR efficiency [25]. For partial sequencing of the VGSC region, BioEdit software was used to manually trace all sequence set to detect polymorphic positions and for ClustalW alignment [26], while haplotype construction/polymorphic analysis was done with DnaSP v5.10 [27]. Sequence data generated from the Akaka-Remo population were compared to sequence sets previously obtained from Kpome [28] and Pahou [24] in southern Benin, and Gounougou [5] in northern Cameroon. In addition, a Neighbour-Joining (NJ) tree was generated using MEG 6.06 after the level of Kst of pairwise genetic differentiation between populations was determined with Dnasp v5.10. Also, Maximum Likelihood (ML) phylogenetic tree was constructed for the VGSC haplotypes in different sample populations with Mega 6.06 using the best constructing model [29].

## Results

### The GSTs and esterase were implicated in pyrethroid and DDT resistance of *An. funestus* (*s.s.*)

**Fig. 1:**
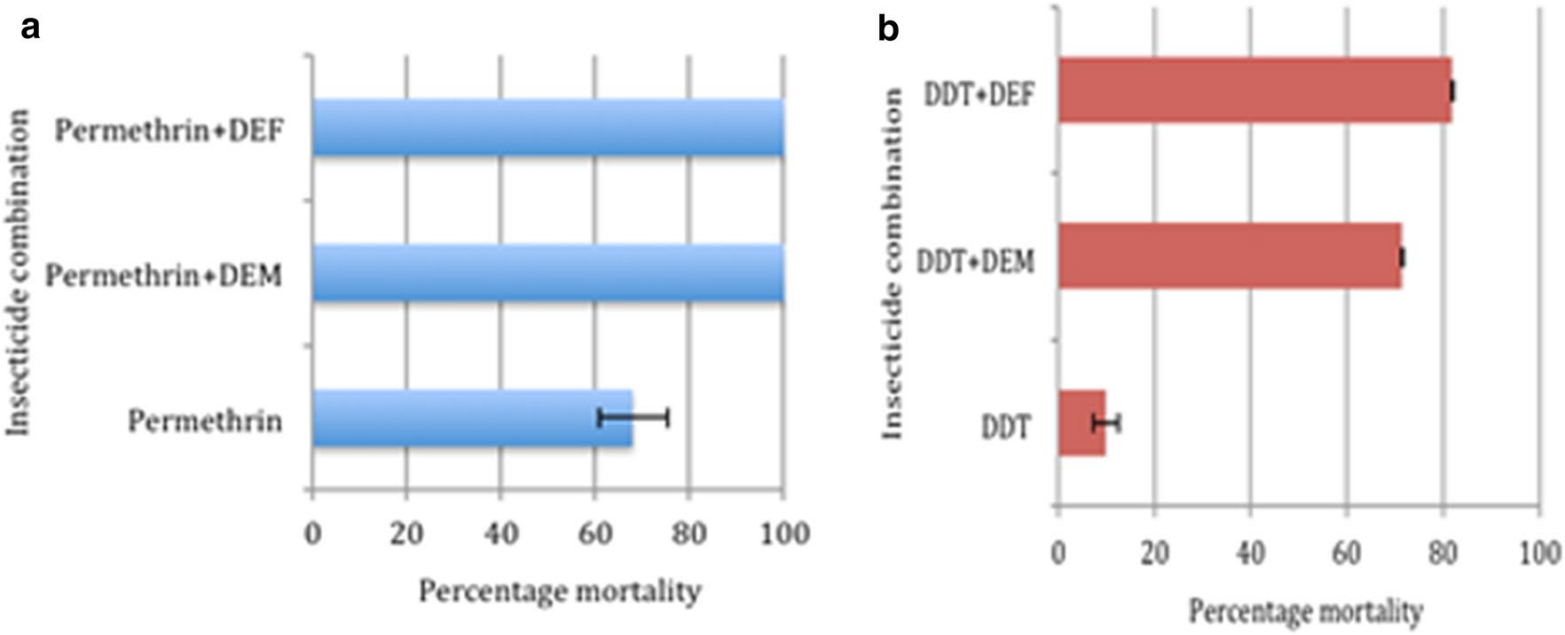
DEM and DEF synergist assessment of permethrin and DDT insecticides

All the 96 blood-fed *An. funestus* (*s.l.*) that oviposited and subjected to the forced egg-laying were morphologically and molecularly identified as *An. funestus* (*s.s.*) as reported in the previous study [1]. Also, the F_1_ mosquitoes generated and tested for insecticide susceptibility were resistant to permethrin (68 ± 5.64%), DDT (10 ± 2.66%) and dieldrin (8 ± 3.24%) as reported in Djouaka et al. [1]. In addition to the role of oxidase in the resistance of this mosquito population as documented in Djouaka et al. [1], esterases and GSTs also showed their involvement in the observed permethrin and DDT resistance in the present study (Fig. 1). *Anopheles funestus* (*s.s.*) showed a 100% mortality with permethrin when pre-exposed to both DEM and DEF, suggesting the implication of GSTs and esterase in permethrin resistance. Similarly, the pre-exposure of mosquitoes to DEM and DEF, and then to DDT resulted in mortalities of 71% and 82%, respectively, which equally suggests the role of GSTs and esterase in DDT resistance.

### Microarray revealed that upregulation of metabolic genes plays a crucial role in permethrin and DDT resistance of *An. funestus* (*s.s.*)

Differentially expressed transcripts were identified in the comparisons of permethrin/DDT resistant samples to controls. Three mosquito populations were analysed for permethrin resistance: Rperm; unexposed control; susceptible FANG, and two populations for DDT resistance: R_DDT_ and susceptible FANG. For comparison analysis, a total of 1536 (798 overexpressed) transcripts were differentially expressed in Rperm-S, 1467 (230 overexpressed) differentially expressed transcripts in Rperm-C, while the R_DDT_-S comparison produced 664 (299 overexpressed) transcripts, as well as 2473 (1093 overexpressed) transcripts in C-S. There was no R_DDT_-C cross analysis due to the high DDT resistance recorded.

### Gene expression profiling showed an association between the upregulation of multiple metabolic genes and permethrin resistance of *An. funestus* (*s.s.*)

**Fig. 2:**
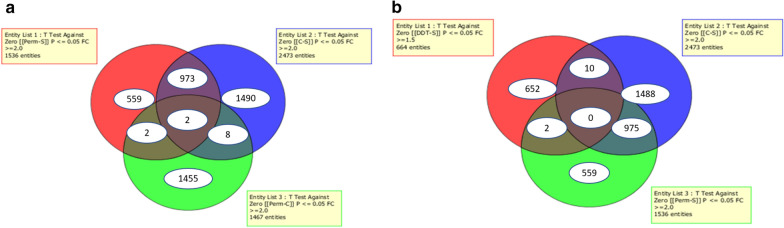
**a** Transcripts differentially expressed in permethrin resistance. **b** Transcripts differentially expressed in the cross-analysis of DDT and permethrin resistance. Venn diagram displays the number of significantly (P ≤ 0.05) up or down-regulated transcripts, and the commonly expressed transcripts (FC ≥ 2) in each comparison

Microarray data analysed with genespring, GX 13.0 software successfully identified commonly expressed transcripts in the different comparisons. There was only one common transcript (Afun000762) overexpressed in all the three comparisons, Rperm-S, C-S and Rperm-C with fold changes (FC) of 5.19, 3.83 and 2.53, respectively (Fig. 2a, Table 2), with no detoxification gene commonly overexpressed in these three comparisons.

**Table 2:** Detoxification genes upregulated in Rperm-S, C-S and Rperm-C

S/N	Transcript name	Gene name	Rperm-S FC	C-S FC	Rperm-C FC	Ortholog in *An. gambiae*	Description
1	CUST_762_PI426302897	Afun000762	5.19	3.83	2.53	AGAP006733	THO complex subunit 4
2	CUST_2376_PI406199772	CD578215.1	17.81	43.40			Cuticle protein
3	CUST_2375_PI406199772	CD578215.1	14.75	36.74			Cuticle protein
4	CUST_15266_PI426302897	Afun015266	2.44	2.88		AGAP010911-PA	Carboxylesterase
5	CUST_10482_PI426302897	Afun010482	18.00	65.91		AGAP008449-PA	Cuticle protein
6	CUST_3752_PI406199772	CD577507.1	3.56	5.87			Cuticle protein
7	CUST_13390_PI426302897	Afun013390	4.11	3.47		AGAP000344-PB	Cuticular protein rr-1
8	CUST_15708_PI406199769	Combined_c8336	2.25				Glucosyl glucuronosyl transferases
9	CUST_7571_PI426302897	Afun007571	2.44			AGAP007920-PA	Glucosyl glucuronosyl transferases
10	CUST_15707_PI406199769	Combined_c8336	2.10				Glucosyl glucuronosyl transferases
11	CUST_8617_PI426302897	Afun008617	2.02			AGAP005222-PB	Cytochrome b561
12	CUST_15122_PI426302897	Afun015122 (GSTU2)	4.25			AGAP003257-PA	Glutathione S-transferase gst
13	CUST_10134_PI426302897	Afun010134	4.75			AGAP006711-PA	Chymotrypsin 1
14	CUST_1378_PI406199798	AGAP001405-RA_2R		2.42		AGAP001405-RA_2R	Short-chain dehydrogenase
15	CUST_484_PI406199788	gb-CYP9J3		2.30			Cytochrome p450
16	CUST_5277_PI406199769	Combined_c2672		2.20			Cuticular protein 97eb
17	CUST_36_PI406199775	COEAE6O		2.15		AGAP002863-PA	Carboxylesterase
18	CUST_5_PI406199775	CYP6AA4		3.49			Cytochrome p450
19	CUST_11060_PI406199798	AGAP011431-RA_3L		2.02		AGAP011431-RA_3L	Trypsin 5
20	CUST_3941_PI406199772	CD577405.1 (GSTS1)		2.00			Glutathione-S-transferase
21	CUST_4873_PI406199772	BU039010.1		2.13			Cytochrome c
22	CUST_8525_PI426302897	Afun008525		20.18		AGAP000047-PA	Cuticle protein rr-1
23	CUST_3943_PI406199772	CD577404.1		2.38			Glutathione S-transferase
24	CUST_3751_PI406199772	CD577507.1		2.32			Cuticle protein
25	CUST_13027_PI406199798	AGAP012291-RA_3L		2.42		AGAP012291-RA_3L	Cytochrome p450
26	CUST_2644_PI406199772	CD578079.1		2.70			Trypsin
27	CUST_2643_PI406199772	CD578079.1		2.98			Trypsin
28	CUST_10994_PI426302897	Afun010994 (CYP6P9b)		14.48		AGAP002867-PA	Cytochrome p450
29	CUST_2814_PI406199769	AGAP004164-RC_glutatathione-S-transferase			2.09	AGAP004164-RC_glutatathione-S-transferase	Glutathione-S-transferase
30	CUST_2245_PI406199798	CYP6P2			2.47		Cytochrome p450
31	CUST_4637_PI406199798	Afun014849			2.03	AGAP011507-PA	Esterase fe4
32	CUST_10523_PI406199798	CYP6P9a			2.92		Cytochrome p450

*Abbreviation*: FC, fold change

### Common transcripts overexpressed in Rperm-S and C-S

There were 445 common transcripts overexpressed in this comparison out of 973 differentially expressed transcripts. Only two of these transcripts are associated with resistance: the cuticle proteins (5 transcripts) and carboxylesterase. Cuticle proteins had higher overexpression: CD578215.1 (Rperm-S, FC = 17.81; C-S, FC = 43.40), CD578215.1 (Rperm-S, FC = 14.75; C-S, FC = 36.74), Afun010482 (Rperm-S, FC = 18; C-S, FC = 65.91), CD577507.1 (Rperm-S, FC = 3.56; C-S, FC = 5.87) and Afun013390 (Rperm-S, FC = 4.11; C-S, FC = 3.47), while carboxylesterase on the other hand produced a relatively lower fold change with Afun015266 (Rperm-S, FC = 2.88; C-S, FC = 2.88) (Table 2).

### Common transcripts overexpressed in Rperm-S

This comparison produced the *GSTu2* (FC = 4.25), a member of the GSTs family. The other resistance-associated genes overexpressed are glucosyl glucuronosyl transferases (3 transcripts) with fold changes of 2.25, 2.44 and 2.10 and chymotrypsin 1 (Afun010134: FC = 4.75). An electron transport trans-membrane protein, cytochrome b561 (Afun008617; FC = 2.20) was also overexpressed.

### Common transcripts overexpressed in C-S

Four transcripts, *CYP9J3* (FC = 2.3), *CYP6P9a* (FC = 2.92), *CYP6P9b* (FC = 14.48) and *CYP6AA4* (FC = 3.49) of the cytochrome P450 monooxygenase were overexpressed in this comparison (Table 2). The *CYP6P9b* had the highest overexpression while the other duplicated gene, *CYP6P9a* that has been implicated in permethrin resistance of different *An. funestus* populations was also overexpressed. Other resistance-associated genes in this group are the short-chain dehydrogenase (AGAP001405-RA_2R, FC = 2.42), carboxylesterase (COEAE6O, FC = 2.15), 3 transcripts of trypsin (AGAP011431-RA_3L, FC = 2.02; CD578079.1, FC = 2.38 and CD578079.1 FC = 2.98), cytochrome C (BU039010.1, FC = 2.13) and 2 transcripts of the cuticle proteins (Afun008525, FC = 20.18 and CD577507.1, FC = 2.32).

### Common transcripts overexpressed in Rperm-C

Two transcripts of the cytochrome P450 genes were overexpressed in the Rperm-C comparison. The *CYP6P9a* (FC = 2.92) and *CYP6P2* (FC = 2.47) were identified to be playing a role in the detoxification of permethrin. The GST gene (AGAP004164-RC, FC = 2.09) was also overexpressed as well as the esterase family, esterase fe4 (Afun014849, FC = 2.03) in this comparison.

### Genes associated with permethrin resistance using RT-PCR technique

**Fig. 3:**
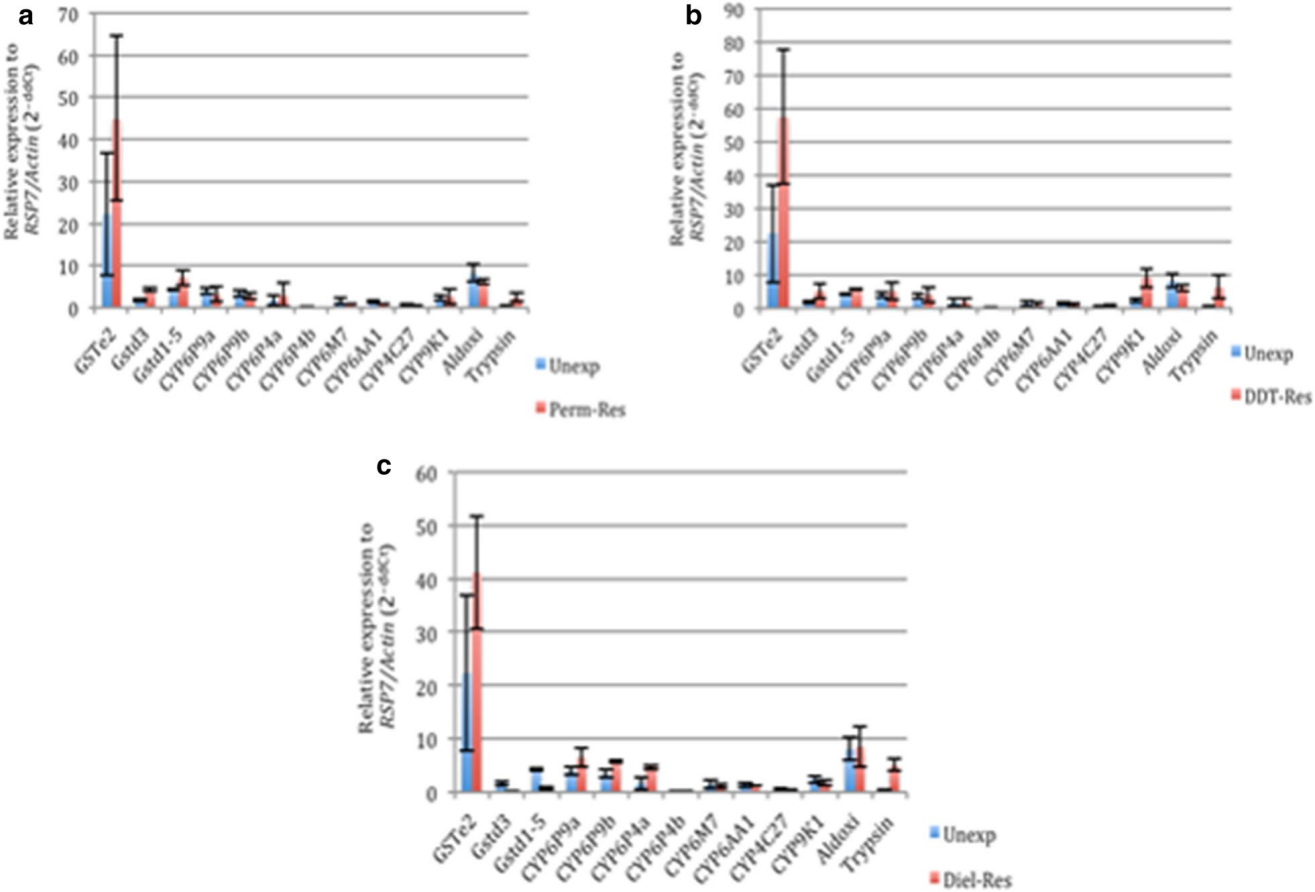
Gene expression analysis of selected candidate genes in *An. funestus* that are resistant to DDT (**a**), permethrin (**b**) and dieldrin (**c**) using the RT-PCR. *Abbreviations*: Unexp, unexposed/control population; perm-Res, permethrin-resistant population; DDT-Res, DDT-resistant population; Diel-Res, dieldrin-resistant population

The *GSTe2* gene was upregulated in permethrin-resistant mosquitoes (FC = 44.89) compared to the unexposed control (FC = 22.34) (Fig. 3a) using the RT-PCR. Both *GSTd3* and *GSTd1-5* were also upregulated in resistant mosquitoes compared to control but with lower folds [*GSTd3*: FC = 4.27 (resistant) *vs* 1.75 (control) and *GSTd1-5*: FC = 7.1 (resistant) *vs* 4.3 (control)] compared to the *GSTe2.* Some cytochrome P450 genes, like the *CYP6P4a* [FC = 2.98 (resistant) *vs* 1.72 (control)] and slightly in *CYP9K1* [FC = 2.66 *vs* 2.44] were upregulated in Rperm over the unexposed control mosquitoes. *Trypsin* was also overexpressed in the resistant (FC = 2.42) compared to the control (0.31).

### Gene expression profiling also revealed that the upregulation of multiple metabolic genes drives DDT resistance of *An. funestus* (*s.s.*)

There was no detoxification gene overexpressed in these different comparisons (R_DDT_-S, C-S and Rperm-S; R_DDT_-S and C-S; R_DDT_-S and Rperm-S; Rperm-S and C-S). However, there were few resistant-associated transcripts (cytochrome P450s, GSTs, carboxylesterase, glucosyl glucuronosyl transferases, chymotrypsin, short-chain dehydrogenase, trypsin and cuticle proteins) identified in other comparisons. There was only one P450 transcript (*CYP6AK1*, FC = 1.69) overexpressed in R_DDT_-S (Table 3). The other transcripts upregulated in this comparison were cuticle protein genes (*AGAP009480-RA_3R*, FC = 1.94; *AGAP003382RA_Cuticular*, FC = 1.84), chymotrypsin 1, (combined_c3760, FC = 2.13), short-chain dehydrogenase (*CD577943.1*, FC = 1.72) and the nucleotide binding protein 2 (*AGAP011997-RA_3L*, FC = 1.64). Transcripts of cytochrome P450s, GSTs are among the detoxification genes that were overexpressed in the C-S comparison. There were four transcripts of the P450 genes in the C-S comparison: *CYP9J3* (FC = 2.30); *CYP4AA4* (FC = 3.49); *CYP6P9b* (FC = 14.48) and *AGAP012291-RA_3L*, (FC = 2.42). There was also an overexpression of the *GSTs1* (FC = 2), a member of the sigma GSTs. The other detoxification genes overexpressed here were the short-chain dehydrogenase, *AGAP001405-RA_2R* (FC = 2.42), carboxylesterase, *COEAE6O* (FC = 2.15), three transcripts of cuticle proteins (*combined_c2672*, FC = 2.2; *Afun008525*, FC = 20.18; *CD577507.1*, FC = 2.32) and three transcripts of trypsin (*AGAP011431-RA_3L*, FC = 2.02; *CD578079.1*, FC = 2.7; *CD578079.1*, FC = 2.98). The cross-analysis between Rperm-S and R_DDT_-S comparison, which was conducted to identify potential cross-resistance genes involved in both DDT and permethrin resistance did not produce any transcript (Fig. 2b, Table 3). However, it produced three transcripts of glucosyl glucuronosyl transferases with fold changes of 2.25 (combined_c8336), 2.44 (Afun007571) and 2.1 (combined_c8336) for the Rperm-S comparison. Also, there was an overexpression of *GSTu2* (FC = 4.25), the transmembrane protein, cytochrome b561 (FC = 2.02) and the digestive enzyme, chemotrypsin 1 (FC = 4.75).

**Table 3:** Detoxification genes upregulated in R_DDT_-S, Rperm-S and C-S

S/N	Transcript name	Gene name	R_DDT-S_ FC	C-S FC	Rperm-S FC	Ortholog in *An. gambiae*	Description
1	CUST_421_PI406199788	gb-CYP6AK1	1.69				Cytochrome P450
2	CUST_7149_PI406199798	AGAP009480-RA_3R	1.94			AGAP009480-RA_3R	Cuticle protein
3	CUST_4284_PI406199798	AGAP003382-RA_Cuticular	1.82			AGAP003382-RA_Cuticular	Cuticle protein
4	CUST_7428_PI406199769	Combined_c3760	2.13				Chymotrypsin 1
5	CUST_2913_PI406199772	CD577943.1	1.72				Short-chain dehydrogenase
6	CUST_13242_PI406199798	AGAP011997-RA_3L	1.64			AGAP011997-RA_3L	Nucleotide binding protein 2
7	CUST_1378_PI406199798	AGAP001405-RA_2R		2.42		AGAP001405-RA_2R	Short-chain dehydrogenase
8	CUST_484_PI406199788	gb-CYP9J3		2.30			Cytochrome p450
9	CUST_5277_PI406199769	Combined_c2672		2.20			Cuticular protein 97eb
10	CUST_36_PI406199775	COEAE6O		2.15		AGAP002863-PA	Carboxylesterase
11	CUST_5_PI406199775	CYP6AA4		3.49			Cytochrome p450
12	CUST_11060_PI406199798	AGAP011431-RA_3L		2.02		AGAP011431-RA_3L	Trypsin 5
13	CUST_3941_PI406199772	CD577405.1 (GSTS1)		2.00			Glutathione S-transferase
14	CUST_8525_PI426302897	Afun008525		20.18		AGAP000047-PA	Cuticle protein rr-1 family
15	CUST_3943_PI406199772	CD577404.1		2.38			Glutathione S-transferase
16	CUST_3751_PI406199772	CD577507.1		2.32			Cuticle protein
17	CUST_13027_PI406199798	AGAP012291-RA_3L		2.42		AGAP012291-RA_3L	Cytochrome p450
18	CUST_2644_PI406199772	CD578079.1		2.70			Trypsin
19	CUST_2643_PI406199772	CD578079.1		2.98			Trypsin
20	CUST_10994_PI426302897	Afun010994 (CYP6P9b)		14.48		AGAP002867-PA	Cytochrome p450
21	CUST_15708_PI406199769	Combined_c8336			2.25		Glucosyl glucuronosyl transferases
22	CUST_7571_PI426302897	Afun007571			2.44	AGAP007920-PA	Glucosyl glucuronosyl transferases
23	CUST_15707_PI406199769	Combined_c8336			2.10		Glucosyl glucuronosyl transferases
24	CUST_8617_PI426302897	Cytochrome b561			2.02	AGAP005222-PB	Cytochrome b561
25	CUST_15122_PI426302897	Afun015122 (GSTU2)			4.25	AGAP003257-PA	Glutathione-S-transferase gst
26	CUST_10134_PI426302897	Afun010134			4.75	AGAP006711-PA	Chymotrypsin 1

*Abbreviation*: FC, fold change

### Genes associated with DDT resistance using RT-PCR technique

**Fig. 4:**
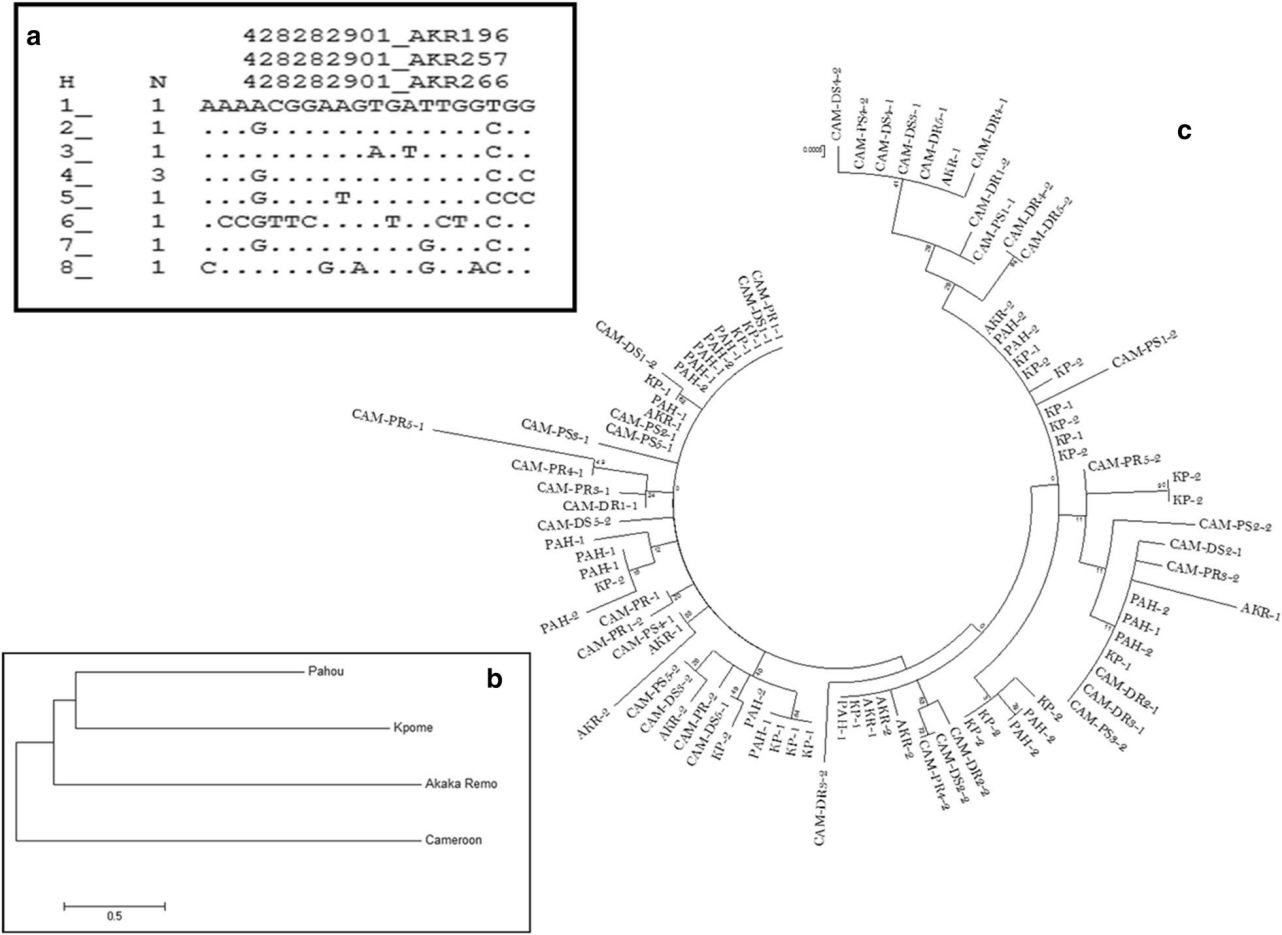
*kdr* polymorphism in *Anopheles funestus* from Akaka-Remo. **a** Schematic representation of haplotypes of Exon20 fragment of the voltage-gated sodium channel gene (VGSC) observed in wild type *An. funestus* from Akaka-Remo. Only the polymorphic sites are shown in the aligned sequence. Dots mean identity with the parent (first) sequence. The column (N) indicates the number of individuals sharing the haplotype. **b** Neighbour joining tree of the VGSC gene between Akaka-Remo, Kpome, Pahou and Cameroon. **c** Maximum likelihood phylogenetic tree of *kdr* in Akaka-Remo, Kpome, Pahou and Cameroon

The *GSTe2* also had the highest expression level in mosquitoes resistant to DDT, producing a fold change of 57.39 in DDT-resistant *vs* FC = 22.34 in unexposed control mosquitoes (Fig. 3b). Also, *GSTd3* expression (FC = 5.1) was almost 3-fold higher in DDT-resistant mosquitoes compared to the control (FC = 1.75), while the expression of *GSTd1-5* in DDT-resistant mosquitoes (FC = 5.51) was just a little more than 1-fold compared to the control (FC = 4.3). The two duplicates (*CYP6P9a* and *CYP6P9b*) cytochrome P450 genes were both overexpressed in the DDT-resistant mosquitoes compared to the unexposed control (Fig. 4b). However, expression was higher in *CYP6P9a* (FC = 5.19) than *CYP6P9b* (FC = 3.91). The *CYP4C27* expression was also slightly higher in the resistant (FC = 0.92) compared to the control (FC = 0.65). The expression of *CYP9K1* was the highest (FC = 9.05) compared to the unexposed control (FC = 2.44) in all of the cytochrome P450 genes analysed. *Trypsin* expression was also highly overexpressed in DDT-resistant (FC = 6.21) compared to the unexposed mosquitoes (FC = 0.31).

### Genes associated with dieldrin resistance using RT-PCR technique

Glutathione S-transferase genes, cytochrome P450 genes, aldehyde oxidase and trypsin were all upregulated in the dieldrin-resistant mosquitoes (Fig. 3c). The *GSTe2* expression was almost 2-fold higher in dieldrin-resistant samples (FC = 41.1) compared to the unexposed control (FC = 22.34). Both the *GSTd3* and *GSTd1-5* genes were downregulated in the dieldrin-resistant mosquitoes compared to the control [*GSTd3*: FC = 0.28 (resistant) *vs* 1.75 (control); *GSTd1-5*: FC = 0.78 (resistant) *vs* 4.30 (control)], whereas both *CYP6P9a* and *CYP6P9b* were overexpressed in dieldrin-resistant samples compared to control. As observed in the DDT-resistant samples, *CYP6P9a* (FC = 6.51) expression was relatively higher than *CYP6P9b* (FC = 5.71). Another P450 gene duplicate, *CYP6P4a* and *CYP6P4b* were overexpressed in dieldrin-resistant mosquitoes compared to control samples. The expression of *CYP6P4a* was higher in dieldrin-resistant (FC = 4.75) compared to control (FC = 1.72) mosquitoes. *Aldehyde oxidase* was slightly overexpressed in dieldrin-resistant samples (FC = 8.58) compared to the unexposed samples (FC = 8.12) while *trypsin* expression was higher in dieldrin-resistant mosquitoes (FC = 5.14) compared to control (FC = 0.31).

### Partial sequencing of the VGSC region showed that the *kdr* mutation is not involved in the insecticide resistance of *An. funestus* (*s.s.*) population

The portion (924 bp) of the VGSC gene spanning intron 19 and the entire exon 20 (207 bp) located on domain II at segment 6 was successfully amplified (PCR) and sequenced in a total of 11 *An. funestus* (*s.s.*) mosquitoes collected from Akaka-Remo. Both L1014F (-TTA- to –TTT-) and L1014S (-TTA- to –TCA-) *kdr* mutations that are common to *An. gambiae* in West and East Africa, respectively, were absent in the sequence analysis. Further analysis with 907-bp sequences obtained and aligned from five individual mosquitoes in this study detected 20 polymorphic sites (887 monomorphic sites) and 8 haplotypes (Fig. 4a, Table 4). Genetic differentiation of this mosquito population analysed using the NJ and ML trees was compared to *An. funestus* (*s.s.*) populations from Benin and Cameroon. Analysis using the NJ tree with respect to geographical distance revealed a similar genetic constitution with the Cameroon population but a higher differentiation than Benin populations (Fig. 4b, Table 4). Also, there was no correlation in the VGSC polymorphisms of the different mosquito populations, which was shown by the lack of clustering of mosquito samples from the same locality in the ML tree (Fig. 4c). The absence of correlation was further supported by the fact that polymorphism did not result into any amino acid change as well as the estimates of Tajima D and Fu and Li D* statistics, which were not statistically significant (Table 4).

**Table 4:** Genetic parameters of VGSC of *Anopheles funestus* from Akaka-Remo compared to Benin and Cameroon populations

Locality	N(2n)	S	Pi (π)	K	h	hd	Syn	Non-syn	D	D*
Akaka-Remo	10	20	0.00524	4.75556	8	0.933	0	0	− 1.54^ns^	− 1.81^ns^
Kpome	22	12	0.00351	2.93939	12	0.909	0	0	− 0.37^ns^	− 0.32^ns^
Pahou	20	10	0.0026	2.17895	12	0.905	0	0	− 0.79^ns^	− 0.96^ns^
Cameroon	40	37	0.00514	4.30128	29	0.977	2	3	− 1.81^ns^	− 2.75^ns^

*Abbreviations*: 2n, number of sequences; S, number of polymorphic sites; π, nucleotide diversity; k, average number of nucleotide difference; h, number of haplotypes; hd, haplotype diversity; syn, synonymous; Non-syn, non-synonymous; D, Tajima’s statistics; D*, Fu and Li’s statistics; ns, not significant

## Discussion

Consistent monitoring of insecticide resistance of malaria vectors through the provision of evidence-based information is key to tackle the insecticide resistance challenges facing malaria elimination in Africa. Understanding the mechanisms of insecticide resistance is imperative to improve insecticide-based resistance management strategies. Therefore, providing the molecular basis of permethrin, DDT and dieldrin resistance in *Anopheles funestus* (*s.s.*), a major malaria vector in Nigeria will fill an important knowledge gap in the quest to fight malaria in the country. This study has successfully shown that metabolic enzymes are playing vital roles in insecticide resistance of *An. funestus* (*s.s.*) at Akaka-Remo. This was observed through the overexpression of several detoxification genes, especially the *GSTe2* and trypsin. Cuticular resistance was also highly implicated in the resistant mosquito population through the overexpression of cuticle proteins in resistant mosquitoes.

### The upregulation of multiple metabolic genes is vital for permethrin resistance in *An. funestus* (*s.s.*)

This study conducted for the first time on this mosquito population revealed that cytochrome P450 genes are slightly involved in the permethrin resistance of this mosquito population. However, it has clearly been previously shown in other *An. funestus* (*s.s.*) populations through metabolism assay [30] that *CYP6P9a* can metabolize both type I and type II pyrethroids. The *CYP6P9a* and *CYP6P9b* duplicates have been associated with pyrethroid resistance in *An. funestus* (*s.s.*) across Africa; cases of resistant Fumoz strain [21], Uganda population [6], in Malawi and Mozambique [10] and also in the Chikwawa district of Malawi [4]. The fact that *CYP6P9a* was only upregulated in Rperm-C comparison in this study could suggest that it might not represent the best possible candidate gene for permethrin resistance of this mosquito population. However, its role in the resistance of this mosquito population is illustrious. The *CYP6P4a, CYP6P2*, *CYP6AA4* and *CYP9J3* are the other potential P450 genes that could be playing a role in permethrin resistance of this mosquito population. In our previous study, the synergist test with PBO gave a 100% recovery of susceptibility as against 68% mortality with only permethrin exposure [1]. This present study has also shown the full involvement of esterase and GSTs in permethrin resistance, with a record of 100% recovery from susceptibility when each of DEM and DEF was combined with permethrin. This observation highlights the comprehensive roles of oxidase, esterase and GSTs in pyrethroid resistance.

The roles of epsilon (*GSTe2*) and delta (*GSTd3* and *GSTd1-5*) GST gene families in permethrin resistance were also clearly displayed. The reason for the absence of these key resistance-associated genes in microarray and its presence in qPCR could simply be as a result of biases in dyes used in microarray experiments [31] and cross-hybridization or non-specific binding of labelled targets to array probes as pointed out earlier [32]. But the fact that specific primers with the RT-PCR experiment could amplify the *GSTe2*, *GSTd3* and *GSTd1-5* genes in permethrin-resistant samples showed their contributions to permethrin resistance of this mosquito population.

The *GSTe2* is a key detoxification gene associated with pyrethroid resistance in *An. funestus* populations. This is not only because of its elevated expression in *An. funestus* when exposed to pyrethroids [10] but also its capability to metabolize more pyrethroids after undergoing genetic modifications [11]. Elevated *GSTe2* expression has previously been implicated in mosquito resistance by acting as a pyrethroid-binding protein and sequestering the insecticide [33] or by protecting mosquitoes against oxidative stress and lipid peroxidation induced by pyrethroid exposure [34]. Also, a partial knockdown of an ortholog *GSTe2* in *Ae. aegypti* led to increasing pyrethroids (deltamethrin) mortality, which also linked *GSTe2* with deltamethrin resistance in this species [35].

Carboxylesterases also showed a great involvement as well as trypsin, indicating that a myriad of several metabolic enzymes is triggered in resistant *An. funestus* (*s.s.*) population that empowers mosquitoes to survive permethrin exposure. Overall, our findings suggest that the *GSTe2* could be playing a great role in insecticide resistance due to its expression level, but the key observation from the pattern of gene expression is that the observed resistance is powered by multiple detoxification processes. The other genes with lower expressions, nevertheless, would have played their own distinct and significant roles to achieve such a high level of insecticide resistance. Some of these metabolic proteins (cytochrome P450, carboxylesterase, trypsin) being a phase I machinery are very important first-line defense enzymes that help in the hydrolysis of target insecticides for the phase II detoxification proteins (GSTs and glucosyl glucuronosyl transferases) to further process phase I metabolites for transport and excretion from the mosquito’s body system. All these, however, need further investigation to examine the distinct role of each gene in resistance.

### DDT resistance of *An. funestus* (*s.s.*) is driven by the upregulation of multiple metabolic genes

The elevated expressions of *GST*_*S*_*1* and *GST*_*U*_*2* suggest their contributions to DDT resistance. The *GST*_*S*_*1* had earlier been shown to be associated with insecticide tolerance in mosquitoes. The sigma family of GST in insects possess a proline/alanine-rich N-terminal extension that helps to aid attachment to the flight muscle [36], which make them structurally effective for metabolism. In addition, *GSTs* show low-level activities with the typical GST substrates, and have high affinity for the lipid peroxidation product 4-hydroxynonenal [37]. As a result, sigma GST duplicates might also play important roles in eliminating the by-products of oxidative stress [38]. Furthermore, the *GSTu2* is yet to be classified into a definite family of GST but it has been shown to share similar phylogeny with the epsilon and delta families of GST [39, 40], which could suggest its role in DDT resistance.

The role of *GSTe2* in DDT resistance is obvious, considering the difference in expression that was recorded in DDT-resistant mosquitoes (FC = 57.39) against the control population (FC = 22.34) with the RT-PCR analysis. The role of *GSTe2* in DDT resistance of *An. funestus* (*s.s.*) has been previously documented [1, 3, 11]. Its elevated expression has been key to its capacity to confer resistance in this major malaria vector [11, 28]. The L119F-GSTe2 mutation, which has now become very common in different resistant *An. funestus* populations in Africa [1, 3, 11] is known to be strengthening the observed phenotypic DDT resistance. Generally, a mosquito carrying this mutation would have an enlarged DDT-binding site, which helps to increase DDT access and metabolism [11]. Leucine (CTT) transformation to phenylalanine (TTT) on position 119 of *GSTe2* gene is predominant and almost getting fixed in *An. funestus* at Akaka-Remo [1]. So, with mutant *GSTe2* dominating the mosquito population, the mosquito would have expressed mainly mutant *GSTe2* enzyme. It is, therefore, possible that the overexpression of *GSTe2* may have been influenced by the high L119F mutation in the mosquito population. However, it is necessary to conduct further investigations to fully establish this possible relationship.

The delta family of the GSTs, *GSTd3* and *GSTd1-5* could also be offering vital metabolic contributions to DDT resistance of this mosquito population due to their higher expressions in resistant compared to control mosquitoes. The P450 genes (*CYP6P9a/b*, *CYP9K1*, *CYP6AK1*, *CYP4C27*, *CYP9J3*, *CYP6AA4* and *CYP6P4a*) also had elevated expressions in DDT-resistant samples compared to the control population but because they have been reported to lack the capacity to metabolise DDT [10], their roles in DDT resistance becomes trivial, although not to be neglected. The digestive enzyme, trypsin could also be playing some certain hydrolytic roles in DDT resistance, which will be clearer with more investigations.

### Dieldrin resistance of *An. funestus* (*s.s.*) was also driven by the upregulation of multiple metabolic genes

The *GSTe2* was observed to be the most overexpressed genes in RT-PCR analysis, suggesting its crucial involvement in dieldrin resistance. Also, the elevated expression recorded with P450 genes (*CYP6P9a/b* and *CYP6P4a*), aldehyde oxidase and trypsin highlight their diverse roles in observed dieldrin resistance. Recently, there was a report of a possible target site resistance mechanism through the high frequency of A296S-RDL mutation in *An. funestus* (*s.s.*) from Akaka-Remo [1]. Overall, it is possible that this mosquito population engage both target site and metabolic mechanisms to withstand the lethal dose (4%) of dieldrin. It is therefore important to conduct further experiments to validate this observation.

### The upregulation of cuticle proteins potentially reduced the penetration of insecticide molecules into *An. funestus* (*s.s.*), contributing to high insecticide resistance

The observed permethrin and DDT resistance in the *An. funestus* (*s.s.*) population was not only driven by detoxifying enzymes but also by cuticular resistance. Cuticle proteins had the highest expression level with the microarray experiments, suggesting their prominent roles in both permethrin and DDT resistance. However, the overexpression of these proteins in unexposed mosquitoes could be the consequence of the high resistance in the mosquito population. This implies that cuticle-based resistance, through the thickening of the mosquito cuticle may be a very important resistance mechanism in *An. funestus* (*s.s.*) population. However, further experiments to determine the specific cuticle proteins and the extent of their impact on insecticide resistance of malaria vectors should be considered. It will also be important to measure the cuticle thickness of resistant mosquitoes as against the susceptible ones to further describe the role of mosquito cuticles in resistance [41, 42].

Nkya et al. [43] highlighted the contributions of other factors, such as anthropogenic and industrial chemical xenobiotics as well as microbial compositions resident in mosquito breeding site, to mosquito resistance. It may also be important to investigate the roles of chemical xenobiotics, especially the common ones that mosquito larvae interact with during feeding and breeding in their breeding sites, and to also explore the contribution of microbiota to *Anopheles* mosquitoes’ resistance; this information will help to further manage and holistically strategize on proper and the most suitable insecticide resistance management tools.

### Insecticide resistance of *An. funestus* (*s.s.*) population is not driven by *kdr* mutations

The knockdown resistance mutation is unlikely to be playing a part in the insecticide resistance observed in the *An. funestus* population. Both L1014F and L1014S mutations that are common to *An. gambiae* in West and East Africa were absent in all the mosquitoes analysed, as the case of other *An. funestus* populations in Africa [5, 6, 8, 24, 28]. The VGSC polymorphisms observed could be as a result of evolution through mutation, genetic drift, migration or natural selection in the mosquito population. These genetic processes could lead to different genome constitution of *An. funestus* in the same population, and consequently could help some mosquitoes to survive insecticide exposure. It is appeasing that the diversity of nucleotide sequence observed might not eventually affect the genetic determinant in this mosquito species. However, the potential role of *kdr* (L1014F/S) in insecticide resistance of *An. funestus* group should be monitored since genetic polymorphism in this mosquito species is always high and it could also help to detect other potential resistance-associated mutations [5, 6].

## Conclusions

This study, which is, to our knowledge, the first to depict the molecular basis of *An. funestus* (*s.s.*) in Nigeria, highlights the vital role of *GSTe2* in permethrin, DDT and dieldrin resistance. The roles played by the cytochrome P450s were also depicted in the multiple metabolic strategies adopted by this mosquito population. In reality, the P450 genes including the carboxylesterase would have acted on toxic insecticides and make them water-soluble before activating the phase II genes (*GSTe2*) to completely render the resulting metabolites harmless. However, further investigations including functional assays will help to ascertain the definite roles of these genes in permethrin and DDT resistance. Reduced insecticide penetration into the mosquito through cuticle thickening could also be in operation and contributed greatly to permethrin and DDT resistance of the *An. funestus* population, which also requires further validation through identifying specific cuticle proteins playing this role in resistant mosquitoes. The resistance mechanisms identified in this mosquito species seem to be peculiar to this mosquito population, so there is need to focus investigations of *An. funestus* populations per region for suitable improvements of insecticide-based malaria control interventions. Exploring other factors that could potentially contribute to insecticide resistance development in mosquito vectors is also very important at this point that insecticide-resistant mosquitoes are rapidly spreading in Africa.

## Data Availability

The datasets supporting the findings herein are included in the article. The DNA data have been deposited in the National Center for Biotechnology Information (NCBI) with the following accession numbers: MT731748, MT731749, MT731750, MT731751, MT731752.

## Abbreviations

GST: glutathione S-transferase
DDT: dichlorodiphenyltrichloroethane
ITNs: insecticide-treated nets
IRS: indoor residual spraying
P450: cytochrome P450 genes
VGSC: voltage-gated sodium channel
*kdr*: knockdown resistance
RT-PCR: reverse transcriptase polymerase chain reactions
LSTM: Liverpool School of Tropical Medicine
DEF: S,S,S-tributylphosphorotrithioate
DEM: diethyl maleate
ESTs: expressed sequence tags
DNA: deoxyribonucleic acid
cDNA: complementary deoxyribonucleic acid
RNA: ribonucleic acid
Rperm: permethrin-resistant
R_DDT_: DDT-resistant
C: control
FANG/S: fully susceptible strain of *An. funestus* mosquito
FC: fold change
*Ald oxi*: aldehyde oxidase
cRNA: complementary ribonucleic acid
NJ: Neighbour-Joining
ML: Maximum Likelihood
PBO: piperonyl butoxide
